# Comparative Diagnostic Yield of Rapid Immunoassay-Based Tests and Nucleic Acid Testing for Transfusion-Transmissible Infections in a Tertiary Care Setting

**DOI:** 10.7759/cureus.115125

**Published:** 2026-08-24

**Authors:** D. K Singh, Ranvijay Singh, Anju Singh, Vinay Tiwari, Snehanshu Shukla, Gaurav Kumar, Mayank Kumar

**Affiliations:** 1 Department of Transfusion Medicine, Rajarshi Dashrath Autonomous State Medical College (RDASMC), Ayodhya, IND; 2 Department of Forensic Medicine, Rajarshi Dashrath Autonomous State Medical College (RDASMC), Ayodhya, IND; 3 Department of Microbiology, Rajarshi Dashrath Autonomous State Medical College (RDASMC), Ayodhya, IND; 4 Department of Pathology, Rajarshi Dashrath Autonomous State Medical College (RDASMC), Ayodhya, IND

**Keywords:** blood screening diagnostics, chemiluminescent immunoassay (clia), enzyme-linked immunosorbent assay (elisa), nucleic acid testing (nat), transfusion-transmissible infections (ttis)

## Abstract

Background: Transfusion-transmissible infections (TTIs), particularly human immunodeficiency virus (HIV), hepatitis B virus (HBV), and hepatitis C virus (HCV), continue to compromise blood safety, especially in high-burden and resource-limited settings. Variability in diagnostic performance across screening modalities contributes to residual risk, particularly during the immunological window period.

Objective: To comparatively evaluate the diagnostic performance and incremental yield of Rapid tests, enzyme-linked immunosorbent assay (ELISA), chemiluminescent immunoassay (CLIA), and nucleic acid testing (NAT) in detecting HIV, HBV, and HCV among blood donors in a tertiary care setting and to identify an optimal screening strategy.

Methods: A cross-sectional analytical study was conducted on 2,153 blood donor samples collected over 12 months from September 2024 to September 2025. Eligible donors were aged 18-60 years. Each sample was tested using rapid card tests, ELISA, CLIA, and NAT. Inclusion required sufficient sample volume and complete testing by all four modalities. Data were analysed using descriptive statistics, focusing on detection rates and inter-modality discrepancies.

Results: Rapid tests showed the lowest detection rates for HIV, hepatitis B surface antigen (HBsAg), and HCV at 0.05%, 0.93%, and 0.19%, respectively. ELISA and CLIA demonstrated higher detection rates, with CLIA showing slightly increased detection for HCV. NAT confirmed fewer positive cases for HIV, HBsAg, and HCV at 0.05%, 1.25%, and 0.14%, respectively, suggesting exclusion of likely false-positive serological results. Incremental analysis showed that ELISA and CLIA detected additional reactive donor samples missed by Rapid tests, while NAT refined diagnostic interpretation by confirming active infection.

Conclusion: Significant variation exists among diagnostic modalities for TTI screening among blood donors. Rapid tests alone are insufficient for reliable screening. ELISA and CLIA improve serological detection, while NAT provides confirmatory accuracy. A tiered screening approach combining serological methods with NAT may enhance transfusion safety in tertiary care blood bank settings.

## Introduction

Transfusion-transmissible infections (TTIs) remain a serious challenge to the safety of blood and transfusion practices in most parts of the world. Human immunodeficiency virus (HIV), hepatitis B virus (HBV), and hepatitis C virus (HCV) are the most clinically relevant, as they are highly prevalent, cause chronic disease, and have the potential to develop long-term complications [1]. Regardless of the screening technologies and regulatory frameworks, there is still a risk of transfusion-associated transmission, especially in low- and middle-income countries where the state of healthcare infrastructure and resource allocation can be insufficient [2]. Most persistence of these infections has been traced to the limited diagnostic ability of available screening modalities, particularly in the initial stages of infection, referred to as the immunological window period, where the traditional serological markers are not necessarily observed [3,4]. 

The blood units transfused each year around the world are in the millions, representing a vital aspect of contemporary treatment in the fields of surgery, obstetrics, cancer, and trauma [5]. Nevertheless, the microbiological safety of blood is also a complicated issue to ensure. In other countries, including India, the burden of TTIs is further exacerbated by a large background rate of HBV and HCV infection in the general population, which increases the chance of the introduction of the disease into the donor pool by asymptomatic carriers [6]. Such an epidemiological situation requires a high degree of sensitivity and specificity in screening plans in order to reduce the residual risk of transmissions [7]. Although the national guidelines require donated blood to be screened against HIV, HBV, and HCV, the differences in testing regimens between institutions have led to inconsistent detection rates and possible loss of transfusion safety [8].

Rapid diagnostic tests are commonly adopted as preliminary screening tools because of their operational benefits, such as low cost, low infrastructure needs, and short turnaround time [9]. Their peculiarities are especially appropriate in the peripheral centres and environments with limited resources [10]. Nevertheless, various studies have indicated their low levels of analytical sensitivity as compared to laboratory-based immunoassays, particularly in the ability to identify low levels of antibody titers in the early phases of infection [11]. This shortage makes it more probable that false-negative outcomes may occur, consequently posing a danger of negative transmission due to transfusion [12]. Therefore, the use of Rapid tests as a sole means of reliance has been debatable as part of an overall blood safety strategy.

ELISA has been the mainstay of serological screening in blood banks. The development of assay design, especially third- and fourth-generation ELISA, has enhanced the sensitivity of diagnoses because it can simultaneously detect antibodies and antigens [13]. These have helped in reducing the number of days in the diagnostic window period. Nevertheless, ELISA cannot be regarded as without limitations since a false-positive reaction can be produced by non-specific binding, cross-reactivity, or technical considerations [14]. This false reactivity may result in futile donor deferral and extra effort on confirmatory testing [15].

Chemiluminescent immunoassay (CLIA) is an automated immunodiagnostic method with high analytical sensitivity, specificity, and throughput. CLIA-based assays have demonstrated strong diagnostic performance for anti-HCV screening, including high sensitivity and specificity in blood donor populations [16]. Moreover, CLIA systems can also be used to support high-throughput testing with less operator dependency, which is a feature appropriate in the tertiary care and high-volume blood bank environment [10]. Comparative evaluations in blood donor screening show that automated chemiluminescent immunoassay systems can provide high sensitivity and specificity for detecting HBV, HCV, and HIV markers; however, diagnostic performance may vary according to the assay platform and the characteristics of the screened population [17].

Nucleic acid testing (NAT) has become an important tool in transfusion medicine as it allows the direct detection of viral RNA or DNA. As compared to serological assays, NAT has the ability to detect infections during the window period when antibodies or antigens have not been produced [8]. This feature would greatly eliminate the remaining risk of TTIs and improve the safety of blood in general [5]. The use of NAT in established healthcare has also shown a significant reduction in transfusion-transmitted diseases. Nevertheless, its application is not more popular in resource-constrained environments because it is expensive, complicated to use, and requires specialised equipment and skilled staff [18].

Previous studies have compared individual screening modalities for HIV, HBV, and HCV in blood donor populations, including Rapid tests versus ELISA and CLIA versus NAT [19,20]. However, relatively limited evidence is available from single institutional cohorts in which Rapid testing, ELISA, CLIA, and NAT are evaluated together on the same donor samples, supporting the need for further comparative assessment of their complementary diagnostic yield. Moreover, it is necessary to put the diagnostic performance in the context of high-burden settings where the cost-effectiveness and operational feasibility are key factors of implementation.

A comparative analysis of these modalities has to be systematic to find the best evidence-based screening modality that is sensitive, specific, cost-effective, and feasible. This strategy is especially applicable to tertiary care centres, which are the referral centres and have the role of ensuring that standards of transfusion safety are high.

Objectives of the study

The present study aimed to compare the detection yield and positivity rates of Rapid tests, ELISA, CLIA, and NAT for HIV, HBV, and HCV among blood donor samples in a tertiary care centre. The study also aimed to describe inter-modality differences in reactive results and assess the incremental detection yield of laboratory-based serological and molecular testing compared with Rapid testing.

## Materials and methods

Study design and setting

An analytical cross-sectional laboratory-based study was carried out in the Blood Bank, Department of Transfusion Medicine, Rajarshi Dashrath Autonomous State Medical College (RDASMC), Ayodhya, which served as the site of data collection, for a period of 12 months from September 2024 to September 2025. The research was planned to compare and contrast the diagnostic efficiency of different screening modalities to detect TTIs. The laboratory runs under standard procedures for donor screening and the infectious disease test, which ensures that there is consistency in sample processing and testing. All operations were done according to institutional transfusion safety and quality assurance. The study design was designed in a way that enabled the sequential comparison of the various methods of diagnosis to be used in the same sample population to reduce inter-sample variation.

Study population and sample size

**Figure 1:**
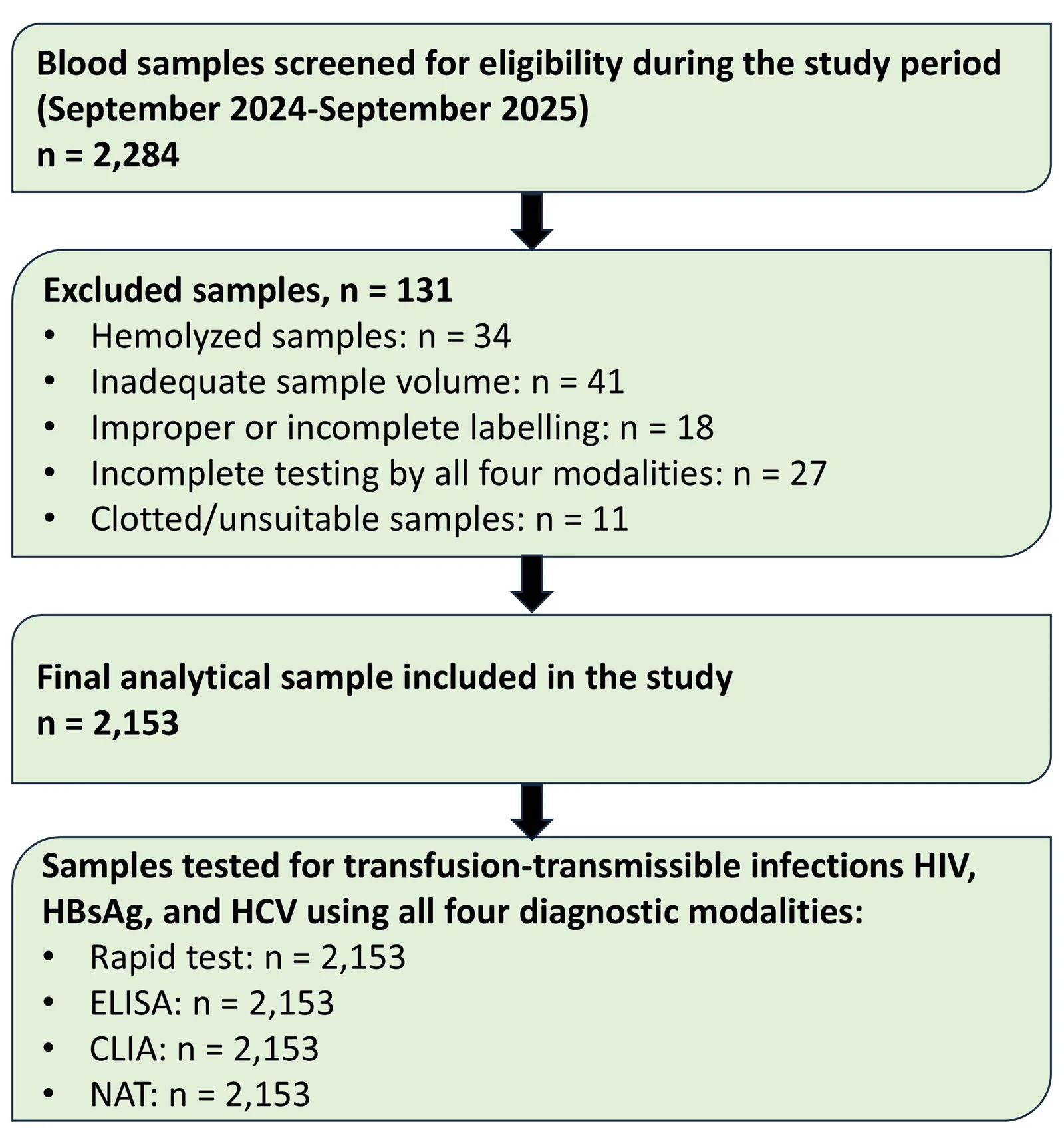
Participant flowchart showing selection of the final analytical sample Created by the authors using Microsoft PowerPoint.

During the 12-month study period, 2,284 blood donor samples were screened for eligibility. Of these, 131 samples were excluded because of hemolysis (n = 34), inadequate sample volume (n = 41), improper or incomplete labelling (n = 18), incomplete testing by all four diagnostic modalities (n = 27), or clotting/other sample unsuitability (n = 11). The remaining 2,153 samples met all eligibility criteria and constituted the final analytical sample. Eligible donors were aged 18-60 years. A consecutive complete enumeration approach was used, in which all eligible samples available during the predefined study period were included. Therefore, no a priori statistical sample-size calculation based on Z, expected prevalence (p), or allowable error (d) was performed. This complete enumeration approach ensured that all eligible samples available during the study period were included, rather than selecting a statistically derived subset. Inclusion in the final analysis required sufficient sample volume and complete testing of the same specimen by Rapid test, ELISA, CLIA, and NAT, permitting direct within-sample comparison across the four diagnostic modalities. Each of the 2,153 included samples underwent testing by all four diagnostic modalities, thereby maintaining a consistent analytical denominator across comparisons. Figure 1 shows the sample-selection process leading to the final analytical sample of 2,153 donors. 

Study duration

**Figure 2:**
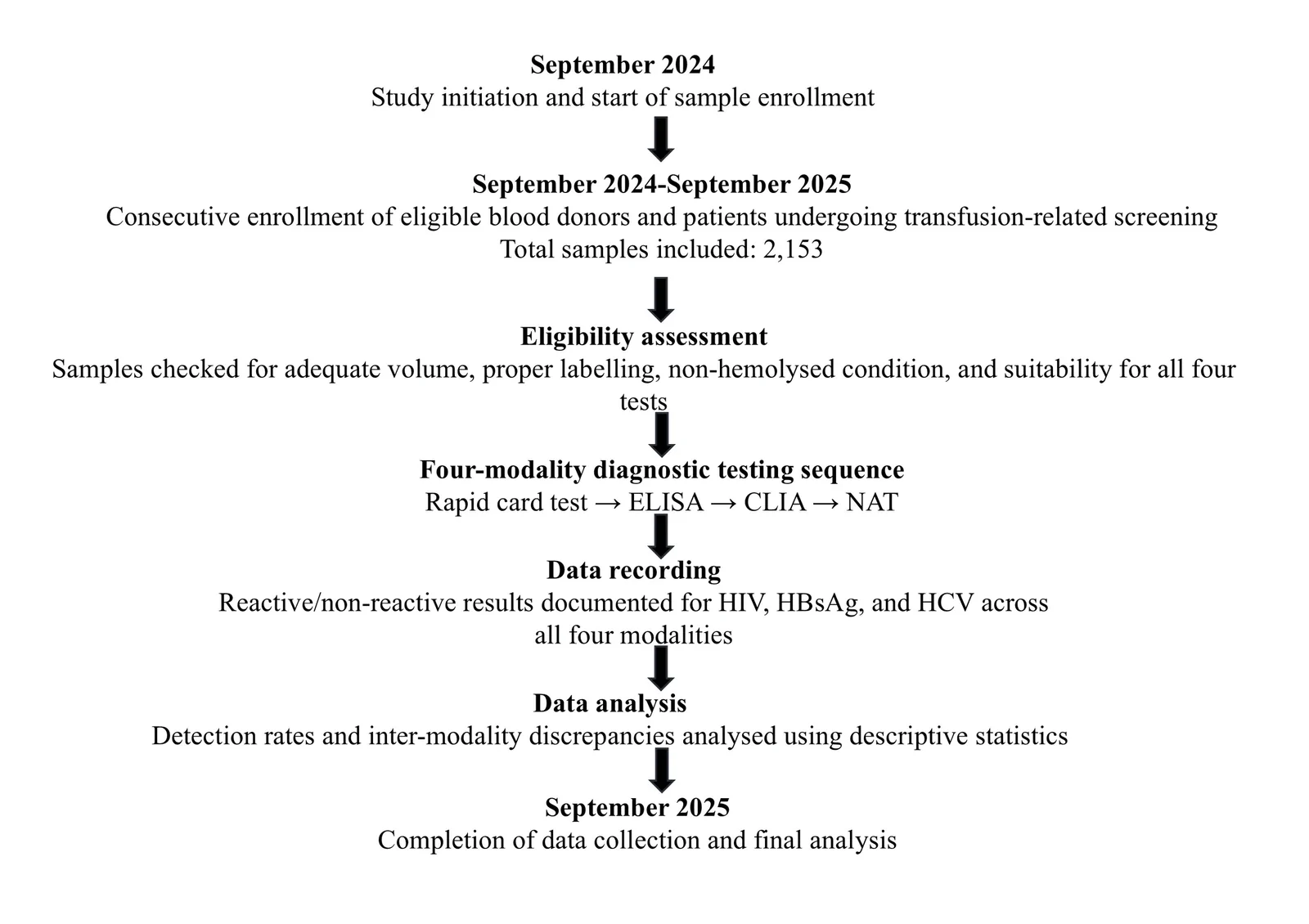
Research timeline and four-modality testing workflow ELISA: enzyme-linked immunosorbent assay; CLIA: chemiluminescent immunoassay; NAT: nucleic acid testing; HIV: human immunodeficiency virus; HBsAg: hepatitis B surface antigen; HCV: hepatitis C virus. Created by the authors using Microsoft PowerPoint.

The study was conducted over a 12-month period from September 2024 to September 2025. This duration was selected to ensure adequate accrual of blood donor samples and to account for possible temporal variation in transfusion-transmissible infection prevalence. Sample collection throughout the study period allowed representation of routine blood bank screening activity across different months. The extended duration also helped reduce seasonal bias and ensured consistency in laboratory procedures and quality control practices. Testing and data recording were performed concurrently to maintain accuracy and minimise documentation errors. Figure 2 shows the overall research timeline, including the data collection window, donor sample enrolment, eligibility assessment, four-modality testing sequence, data recording, and final analysis.

Sample collection and handling

Blood donors were gathered using normal aseptic methods after screening of the donors. Adequate labelling of samples using the identification number was done to ascertain traceability during testing. Specimens collected were treated as per the routine protocols of blood banks, such as proper storage before analysis. The handling was done carefully to prevent hemolysis and contamination. Only those samples that were big enough to be subjected to all the testing modalities were considered. Following standard operating procedures ensured the integrity of the samples, and the results of diagnostic testing could be reliable and reproducible regardless of the testing platform used.

Diagnostic testing strategy

**Figure 3:**
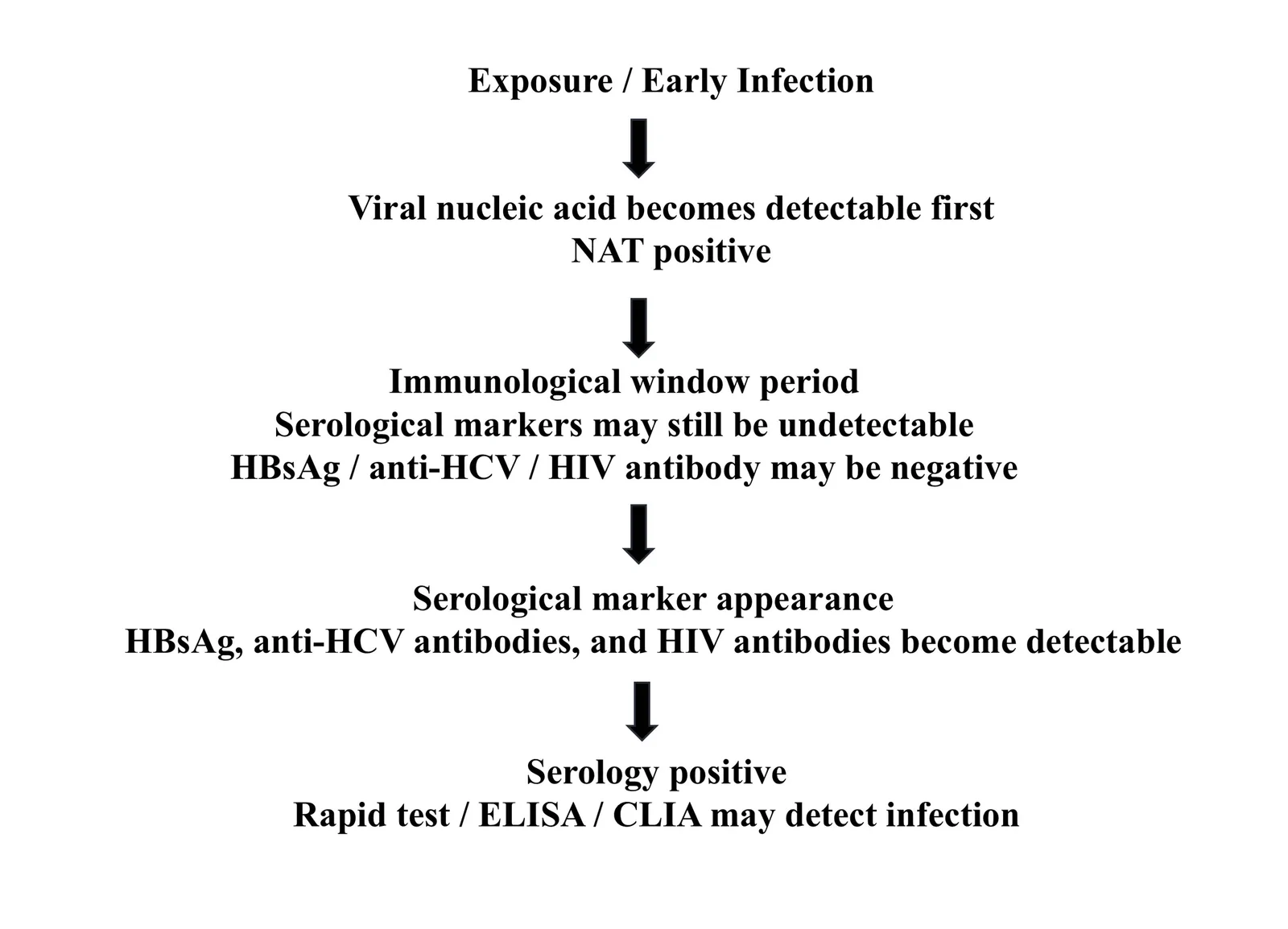
Immunological window period and detection of transfusion-transmissible infections NAT: nucleic acid testing; HBsAg: hepatitis B surface antigen; HCV: hepatitis C virus; HIV: human immunodeficiency virus; ELISA: enzyme-linked immunosorbent assay; CLIA: chemiluminescent immunoassay. Created by the authors using Microsoft PowerPoint.

Each sample was sequentially screened for HIV, hepatitis B surface antigen (HBsAg), and hepatitis C virus (HCV) using four diagnostic modalities: Rapid card tests, enzyme-linked immunosorbent assay (ELISA), chemiluminescent immunoassay (CLIA), and nucleic acid testing (NAT). Initial screening was performed using Rapid card tests, followed by serological assessment with ELISA and CLIA, while NAT was used for molecular detection of viral nucleic acid to establish active infection. All assays were conducted according to manufacturer guidelines and institutional standard operating procedures. Rapid card test results were interpreted visually as reactive or non-reactive, ELISA and CLIA results were interpreted using assay-specific cut-off/index values, and NAT results were interpreted as detected or not detected according to platform-defined criteria. No independent clinical scoring system or grading scale was used in this study. The diagnostic principle underlying NAT-based early detection during the immunological window period is illustrated schematically in Figure 3.

Inclusion and exclusion criteria

Blood donors were included if they were obtained from eligible blood donors or patients undergoing transfusion-related screening, had sufficient volume to perform all four diagnostic modalities, and had complete results available for HIV, HBsAg, and HCV using Rapid card test, ELISA, CLIA, and NAT. Hemolysed samples, improperly labelled specimens, samples with inadequate volume, and samples tested by fewer than all four diagnostic modalities were excluded. These criteria were applied to maintain methodological consistency, reduce selection bias, and ensure valid comparison of diagnostic performance across all testing platforms.

Statistical analysis

Descriptive statistical analysis was performed using Microsoft Excel 2021 (Microsoft Corporation, Redmond, WA, USA). Data were summarized as frequencies, percentages, and 95% confidence intervals for positivity proportions across each testing modality. Incremental detection rates of Rapid tests, ELISA, CLIA, and NAT were compared descriptively to assess differences in detection yield. Because the study did not include a predefined diagnostic reference standard or paired diagnostic contingency data suitable for formal accuracy analysis, sensitivity, specificity, predictive values, and inferential diagnostic-accuracy comparisons were not calculated.

## Results

Overall donor characteristics

The 2153 blood donors underwent Rapid tests, ELISA, CLIA, and NAT to test for HIV, hepatitis B surface antigen (HBsAg), and hepatitis C virus (HCV). Each of the donors was fully tested in the four modalities, which allowed the donors to be compared directly in diagnostic performance. The data set portrays the common conditions of screening the blood bank routines in a tertiary care unit. The general seroreactivity levels were low in all the markers, which is in line with donor-based populations. Nonetheless, intermodal differences in detection were found, indicating differences in analytical sensitivity and specificity. These differences laid the groundwork for later comparisons of diagnostic yield.

Demographic distribution

Gender-Wise Distribution

Out of 2153 donors, 1380 (64.1%) were males, and 773 (35.9%) were females. The percentage of seroreactive cases was higher in males in all three infections. This spread is in line with higher risks of exposure in male populations, which include occupational mobility and behavioural factors. The gender gap indicates the need to address the issue with specific screening and awareness measures. Table 1 provides the gender distribution of the study population, with a majority of the participants being male.

**Table 1:** Gender-wise distribution of study population Data are presented as frequency and percentage, n (%). No inferential statistical test was applied. n: number of cases.

Gender	Number of Cases	n (%)
Male	1380	64.1%
Female	773	35.9%

Age-Wise Distribution

The analysis by age showed that the majority of donors were clustered in the 21-30 years (28.6%) and 31-40 years (25.1%) age groups. These age groups showed the greatest seropositivity, and these are the age groups that are considered to be sexually active and economically productive. It was found to be less prevalent in people under the age of 20 years and over 50 years. This is in line with familiar epidemiological trends of TTIs. The results underscore the importance of dedicated screening procedures in the high-risk age group to minimise the risk of transmission in transfusion environments. Table 2 shows the age-based distribution of the study population, whereby the age brackets of 21-30 and 31-40 years showed the highest percentage.

**Table 2:** Age-wise distribution of study population Data are presented as frequency and percentage, n (%). No inferential statistical test was applied. n: number of cases.

Age Group (Years)	Number of Cases	n (%)
<20	210	9.8%
21-30	615	28.6%
31-40	540	25.1%
41-50	425	19.7%
>50	363	16.8%

Rapid test results

Rapid card tests showed the lowest detection rates for all three infections. HIV was reactive in 1 sample (0.05%), HBsAg in 20 samples (0.93%), and HCV in 4 samples (0.19%). Most donor samples were non-reactive across all markers, with non-reactivity exceeding 99% for HIV, HBsAg, and HCV. The low detection rate suggests limited analytical sensitivity of rapid card tests, particularly during early infection or low-level serological reactivity. These findings indicate that rapid tests may underestimate true seroreactivity when used alone. The results support caution against their independent use for blood donor screening, particularly in settings where early and accurate detection is essential for transfusion safety.

ELISA and CLIA Results

ELISA showed higher detection than rapid card tests, identifying 5 HIV-reactive samples (0.23%), 28 HBsAg-reactive samples (1.30%), and 6 HCV-reactive samples (0.28%). CLIA showed comparable or slightly higher detection, with 4 HIV-reactive samples (0.19%), 28 HBsAg-reactive samples (1.30%), and 7 HCV-reactive samples (0.32%). Both modalities improved the detection of seroreactive donor samples, indicating greater analytical sensitivity than rapid card testing. CLIA showed a slight incremental yield over ELISA for HCV, which may be related to chemiluminescent signal amplification and automated detection. Differences between serological assays and NAT indicate the possibility of false-positive serological reactivity. These findings support the utility of ELISA and CLIA as routine serological screening methods in blood donor screening.

NAT Results

Nucleic acid testing (NAT) was used to detect active viral infection through identification of viral nucleic acid. NAT detected HIV in 1 sample (0.05%), HBsAg-associated HBV infection in 27 samples (1.25%), and HCV in 3 samples (0.14%). NAT showed lower positivity than ELISA and CLIA for HIV and HCV and slightly lower positivity than serological assays for HBsAg, suggesting exclusion of likely false-positive serological results. Since NAT detects viral RNA or DNA directly, it can identify true viraemia and may detect infection during the immunological window period before serological markers become detectable. These findings demonstrate the confirmatory value of NAT in improving diagnostic accuracy, although its routine implementation may be limited by cost, infrastructure requirements, and the need for trained personnel.

Comparative seroreactivity across modalities

Comparative analysis showed a progressive increase in detection from rapid card tests to ELISA and CLIA, followed by refinement of results through NAT. Rapid tests detected the fewest reactive donor samples, whereas ELISA and CLIA identified additional seroreactive samples across HIV, HBsAg, and HCV. NAT reduced the number of positive results for some markers by confirming active infection and excluding likely false-positive serological reactivity. The strongest incremental detection pattern was observed for HBsAg and HCV. This trend highlights the complementary roles of serological and molecular testing in blood donor screening. ELISA and CLIA improve screening sensitivity, whereas NAT strengthens confirmatory accuracy and supports safer transfusion practice.

Infection-specific detection patterns

HIV Detection

Rapid card testing identified 1 HIV-reactive sample (0.05%). ELISA detected 5 HIV-reactive samples (0.23%), while CLIA detected 4 HIV-reactive samples (0.19%). NAT confirmed HIV infection in 1 sample (0.05%). The lower NAT-confirmed positivity compared with ELISA and CLIA suggests that the additional seroreactive results may represent false-positive serological reactivity. This finding highlights the importance of molecular confirmation, particularly in low-prevalence blood donor screening settings where even small differences in assay specificity can affect interpretation and donor deferral decisions.

HBsAg Detection

Rapid card tests identified 20 HBsAg-reactive samples (0.93%). ELISA and CLIA each detected 28 HBsAg-reactive samples (1.30%). NAT confirmed 27 samples (1.25%), indicating close agreement between the laboratory-based serological assays and molecular testing. The difference of one sample between ELISA/CLIA and NAT suggests possible false-positive serological reactivity. The overall similarity among ELISA, CLIA, and NAT indicates relatively consistent detection of HBV infection compared with HIV and HCV in this donor population.

HCV Detection

Rapid card testing identified 4 HCV-reactive samples (0.19%). ELISA detected 6 HCV-reactive samples (0.28%), while CLIA detected 7 HCV-reactive samples (0.32%). NAT confirmed HCV infection in 3 samples (0.14%). The higher serological reactivity compared with NAT suggests possible false-positive serological results, especially with CLIA, which showed the highest detection rate. This pattern indicates that CLIA may improve serological detection, but NAT remains important for confirming active HCV infection and reducing misclassification.

Incremental Diagnostic Yield

Incremental comparison showed that ELISA detected additional reactive samples compared with rapid card tests, including 4 additional HIV-reactive samples, 8 additional HBsAg-reactive samples, and 2 additional HCV-reactive samples. CLIA showed similar detection to ELISA for HBsAg, detected one fewer HIV-reactive sample than ELISA, and identified one additional HCV-reactive sample compared with ELISA. NAT detected fewer positive samples than CLIA for HIV, HBsAg, and HCV, which supports its role in confirming active viral infection and excluding likely false-positive serological reactivity.

Table 3 presents the total number and percentage of reactive cases across all diagnostic modalities.

**Table 3:** Detection of reactive cases across diagnostic modalities Data are presented as the number of reactive cases with percentage positivity, n (%). No inferential statistical test was applied. ELISA: Enzyme-linked immunosorbent assay; CLIA: Chemiluminescent immunoassay; NAT: Nucleic acid testing; HBsAg: Hepatitis B surface antigen; HCV: Hepatitis C virus; HIV: Human immunodeficiency virus; CI: Confidence interval.

Marker	Rapid n (%) [95% CI]	ELISA n (%) [95% CI]	CLIA n (%) [95% CI]	NAT n (%) [95% CI]
HIV	1 (0.05) [0.01–0.26]	5 (0.23) [0.10–0.54]	4 (0.19) [0.07–0.48]	1 (0.05) [0.01–0.26]
HBsAg	20 (0.93) [0.60–1.43]	28 (1.30) [0.90–1.87]	28 (1.30) [0.90–1.87]	27 (1.25) [0.86–1.82]
HCV	4 (0.19) [0.07–0.48]	6 (0.28) [0.13–0.61]	7 (0.33) [0.16–0.67]	3 (0.14) [0.05–0.41]

## Discussion

The current study demonstrated differences in positivity rates and detection yield among Rapid tests, ELISA, CLIA, and NAT for HIV, HBV, and HCV among blood donor samples. Rapid tests reported the fewest reactive cases across all markers, which is consistent with their reliance on visually detectable antigen-antibody reactions and lower analytical sensitivity in comparison with laboratory-based immunoassays. This finding is clinically relevant because screening assays used in blood banks must detect infections at low antigen or antibody concentrations, particularly in blood donors with early infection or low-level serological reactivity. Comparative evaluations have shown that the diagnostic sensitivity of rapid assays for HIV, HBV, and HCV can vary by test kit and may be lower than that of ELISA-based assays, with false-negative results remaining a concern in blood screening [14,15]. ELISA detected more reactive cases across all infections, suggesting improved detection through standardised antigen-antibody interaction and assay amplification. CLIA demonstrated comparable or slightly higher detection, especially for HCV, which may be related to chemiluminescent signal amplification and improved analytical performance.

Of particular importance is the distinction between serological methods such as ELISA and CLIA and molecular confirmation by NAT. NAT registered fewer reactive cases for HCV and slightly fewer cases for HBsAg than serological assays, supporting its role in confirming active infection through direct detection of viral nucleic acid. This reduction in apparent positivity suggests that some serological results may represent false-positive reactivity caused by cross-reactivity, non-specific binding, or technical factors. In HIV, the similarity between Rapid testing and NAT indicated concordance in confirmed cases, whereas the additional ELISA and CLIA reactivity was not confirmed by molecular testing. These findings reflect the expected difference between immunological screening and molecular confirmation in donor screening programs.

The discrepancy between serological reactivity and NAT confirmation also highlights the difference between screening sensitivity and confirmatory specificity. Serological platforms such as ELISA and CLIA are designed to identify antibody or antigen reactivity, whereas NAT directly detects viral nucleic acid and is more closely associated with active viraemia. Comparative studies of NAT, CLIA, and rapid testing have reported similar findings, with NAT improving diagnostic confidence by confirming true infections and reducing residual transfusion risk [5,6]. This is particularly important in transfusion medicine because even a small number of missed infections can have substantial clinical and public health consequences. The incremental detection pattern observed across modalities further supports the complementary role of these tests. ELISA and CLIA identified additional reactive cases compared with Rapid tests, while NAT refined the results by confirming true positivity and reducing likely false-positive serological reactivity. This stratified pattern does not necessarily indicate inconsistency among modalities but reflects the expected diagnostic sequence from initial screening to confirmatory verification. Such a layered approach is useful in blood bank settings where both false-negative and false-positive results have important consequences, including infectious risk to recipients, unnecessary donor deferral, and increased confirmatory testing workload.

The identified difference in diagnostic yield carries serious consequences for transfusion medicine and public health. Blood safety depends on accurate identification of infectious agents with minimal false-negative and false-positive results. The reduced detection by Rapid tests indicates that reliance on a single screening modality could result in under-detection of infection, especially during early stages [15]. ELISA and CLIA remain useful for routine blood donor screening programs because they provide higher detection rates and are more suitable for organised laboratory settings, particularly in high donor-volume centres. NAT is an important diagnostic tool because it confirms active infection, minimises false-positive outcomes, and detects viral nucleic acid during the immunological window period, which cannot be reliably identified by conventional serology-only assays [8,19]. Its use is limited by cost, infrastructure requirements, equipment needs, and trained personnel, making selective or tiered implementation more practical in resource-limited settings.

The public health importance of improved screening is supported by studies reporting persistent TTI burden among blood donors in different regions, including Africa, India, and other high-burden settings [12]. These studies show that background prevalence, donor characteristics, regional epidemiology, and screening infrastructure influence the residual risk of transfusion-transmissible infections. In settings where HBV and HCV remain prevalent, higher-sensitivity platforms may reduce missed infections and strengthen haemovigilance. The present findings support the need for screening algorithms that are adapted to institutional capacity and local disease burden rather than relying on a single diagnostic modality. An integrated approach to blood safety consists of serological assessment combined with molecular confirmation as part of a tiered screening strategy. Rapid tests may retain value in emergency or peripheral settings because of their low cost, short turnaround time, and minimal infrastructure requirements. ELISA or CLIA should be preferred for routine laboratory-based screening where infrastructure permits. NAT can be used for confirmation, discrepant serological findings, reactive donor samples, or centres aiming to reduce window-period transmission. This approach balances diagnostic sensitivity, operational feasibility, and cost constraints while strengthening transfusion safety.

The results of the current study are consistent with previous comparative evaluations of screening methods for HIV, HBV, and HCV. Studies assessing rapid diagnostic tests against ELISA have shown that the sensitivity of some rapid assays may be lower and can vary substantially between kits, particularly for low-reactivity samples [20,21]. These limitations are relevant to blood screening because false-negative rapid-test results may compromise transfusion safety. ELISA-based assays generally demonstrate high analytical performance for HIV, HBV, and HCV and remain suitable for routine laboratory-based blood screening [22]. The comparable performance of ELISA and CLIA in this study is also supported by evidence showing that CLIA can provide improved analytical sensitivity and may be particularly useful for HCV detection [23]. This is consistent with the slightly higher HCV detection observed with CLIA in the present study.

NAT has been widely recognised for its role in confirming true infections, reducing false-positive results, and decreasing residual transfusion risk [19]. Its ability to detect viral nucleic acid before serological markers become detectable makes it especially valuable during the window period. Incremental NAT yield has also been reported in high-burden settings, supporting its role as an important adjunct to serological testing [24]. The present findings are consistent with recent research suggesting that ELISA and CLIA outperform Rapid tests in infection detection, while NAT provides the most conclusive confirmation of active infection [13,25]. The agreement between the current findings and existing national and international evidence strengthens the validity of the observed diagnostic pattern.

The findings support a multimodal screening model for transfusion-transmissible infections among blood donors. Rapid testing alone appears insufficient for reliable blood screening because of lower detection across HIV, HBsAg, and HCV. ELISA and CLIA improve diagnostic yield and are appropriate for routine serological screening in organised blood bank settings. NAT provides confirmatory value by detecting active viral nucleic acid and reducing likely false-positive serological results. A tiered strategy using ELISA or CLIA for routine screening with NAT for confirmation or discrepant/reactive donor samples may offer a practical and evidence-based approach to improving transfusion safety.

Limitations and future recommendations

The current study has certain limitations. It was conducted at a single tertiary care centre, which may limit the generalizability of the findings to other healthcare settings with different donor profiles, infection prevalence rates, laboratory infrastructure, and resource availability. The analysis was primarily descriptive, and formal diagnostic-accuracy measures, including sensitivity, specificity, positive predictive value, negative predictive value, inter-assay agreement, and repeatability, could not be calculated because the study did not include a predefined reference standard, paired diagnostic contingency data, or replicate testing. ELISA was therefore not retrospectively designated as a gold-standard comparator. A formal cost-effectiveness analysis was also not performed, although this is relevant for large-scale implementation of advanced screening modalities such as NAT. The study was restricted to eligible blood donors aged 18-60 years; therefore, the findings may not be directly applicable to paediatric populations, elderly individuals, or non-donor clinical screening groups.

Future studies should include multicentric research across diverse blood donor populations to improve external validity and applicability. Further prospective research should incorporate an appropriate reference standard, paired sample-level diagnostic data, and repeat testing to permit formal assessment of sensitivity, specificity, predictive values, inter-assay agreement, diagnostic validity, and repeatability across ELISA, CLIA, and NAT. Further research should also incorporate cost-benefit analysis to support evidence-based screening policies. Evaluation of multiplex NAT platforms, individual donor NAT, point-of-care molecular assays, and automated high-throughput testing systems may help define scalable and resource-sensitive screening algorithms. Longitudinal studies are also recommended to assess the real-world impact of combined serological and molecular screening strategies on transfusion safety.

## Conclusions

The current study reveals clear disparities in the diagnostic yield of Rapid tests, ELISA, CLIA, and Nucleic Acid Testing (NAT) for detecting transfusion-transmissible infections among blood donors. Rapid card tests demonstrated the lowest detection across all markers, indicating that they should not be used as independent screening tools for blood donor screening. ELISA and CLIA detected more seroreactive donor samples, reflecting improved analytical sensitivity and broader applicability in routine laboratory-based blood screening. CLIA showed a slight incremental increase in detection, particularly for HCV, suggesting its usefulness in selected screening settings. NAT showed fewer positive results than some serological assays because it confirmed active infection and excluded likely false-positive serological reactivity, supporting its role as a confirmatory modality. The variation in results across testing platforms emphasises the complementary value of serological and molecular methods. A combined screening approach using ELISA or CLIA with NAT provides a systematic and effective strategy for improving infection detection, diagnostic consistency, and blood screening accuracy in a tertiary care blood bank setting.
